# Assessment of parental perception of malaria vaccine in Tanzania

**DOI:** 10.1186/s12936-015-0889-7

**Published:** 2015-09-17

**Authors:** Idda Romore, Ali Mohamed Ali, Innocent Semali, Hassan Mshinda, Marcel Tanner, Salim Abdulla

**Affiliations:** Swiss Tropical and Public health Institute, Socinstrasse 57, Postfach, 4002 Basel, Switzerland; University of Basel, Basel, Switzerland; Ifakara Health Institute (IHI), P.O. Box 78373, Dar Es Salaam, Tanzania; Muhimbili University of Health and Allied Science (MUHAS), P.O. Box 65015, Dar Es Salaam, Tanzania; Commision for Science and Technology (COSTEC), P.O. Box 4302, Dar Es Salaam, Tanzania

**Keywords:** Malaria vaccine RTS,S, Parental perception, Willingness, Awareness

## Abstract

**Background:**

Clinical trials of the RTS,S malaria vaccine have completed Phase III and the vaccine is on track for registration. Before making decisions about implementation, it is essential to prepare the ground for introducing the vaccine by assessing awareness and willingness to use malaria vaccines and to provide policy makers with evidence-based information on the best strategies to engage communities to manage the introduction of malaria vaccine in Tanzania.

**Methods:**

In November 2011, as part of a large cross-sectional study of all 23 regions of Tanzania (mainland Tanzania and Zanzibar) was conducted during Tanzanian Integrated Measles Campaign (IMC) survey. In this study, the variables of interests were awareness and willingness to use a malaria vaccine. The main outcome measure was willingness to use a malaria vaccine. Logistic regression was used to examine the influence of predictive factors.

**Results:**

A representative sample of 5502 (out of 6210) women, aged 18 years or older and with children under 11 months old, was selected to participate, using random sampling probability. Awareness of the forthcoming malaria vaccine, 11.8 % of participants in mainland Tanzania responded affirmatively, compared to 3.4 % in Zanzibar (*p* value <0.0001). 94.5 % of all respondents were willing to vaccinate their children against malaria, with a slight difference between mainland Tanzania (94.3 %) and Zanzibar (96.8 %) (p value = 0.0167).

**Conclusions:**

Although mothers had low awareness and high willingness to use malaria vaccine, still availability of malaria vaccine RTS,S will compliment other existing malaria interventions and it will be implemented through the Immunization, Vaccines and Biologicals (IVB) programme (formerly EPI). The information generated from this study can aid policy makers in planning and setting priorities for introducing and implementing the malaria vaccine.

**Electronic supplementary material:**

The online version of this article (doi:10.1186/s12936-015-0889-7) contains supplementary material, which is available to authorized users.

## Background

Malaria still remains a significant public health problem in sub-Saharan Africa, including Tanzania, accounting for 10 % of the burden of disease [1]. Recently, technical innovations to control malaria have contributed to a decline in the malaria burden, but the disease remains a significant threat due to persistent enabling environments, poverty and fragile health systems [1]. Therefore, additional strategies are needed to ensure a combination of interventions that target the various phases of the malaria life cycle, including malaria vaccination [2]. Vaccines are considered cost-effective interventions to reduce and eliminate burden of infectious diseases [3–9].

There are on-going efforts to deliver malaria vaccines as a means to achieving elimination of malaria. Malaria vaccine RTS,S is the most advanced candidate to undergo large scale Phase III evaluation in Africa. It has been tested in eight countries with varying degrees of prevention of malaria transmission. The study sites included: Nanoro in Burkina Faso; Kintampo and Agogo in Ghana; Lambarene in Gabon; Manhica in Mozambique; Lilongwe in Malawi; Kilifi, Siaya and Kombewa in Kenya; and Bagamoyo and Korogwe in Tanzania [10]. Furthermore, phase II and III clinical trials of RTS,S showed that the vaccine reduced the episodes of malaria among young children and infants in malaria endemic areas by half [10–13]. Upon completion of the clinical trials, policy makers will need to make evidence-based decision on the best ways to engage communities to facilitate introduction of malaria vaccine in the national health systems using Tanzania as a case study. The availability of RTS,S will contribute to a multi-intervention approach to controlling malaria that currently uses long-lasting insecticide-treated nets (LLITNs), insecticide-treated nets (ITNs), indoor residual spraying (IRS), and other means of disease reduction and effective drug treatment [10].

Studies on vaccine adherence interventions and acceptance of vaccines recommended use of strategies that will enhance positive community knowledge and perceptions on vaccine effectiveness [14, 15]. Effectiveness of vaccines rely on both clinical efficacy and on a community’s perceptions [8]. During vaccine promotion lack of community support due to poor knowledge and perceptions resulted into poor community uptake while others reject vaccines [16]. In such contexts aligning stakeholders is an important input as was suggested in the network analysis to examine decision-making space in Nigeria [17, 18]. Another similar experiences was the community refection of deworming programme in Ghana [19].

Whereas Tanzania shares similar social cultural and economic contexts to those countries mentioned above there is a high likelihood that new or even current vaccines can be similarly rejected and thus undermining efforts to adopt new technologies to address the high burden of disease. Therefore, it is crucial that community awareness of and willingness to use the malaria vaccine as well as community perceptions of its likely impact are well understood and used to highlight any community-based issues that need to be considered during policy deliberation and intervention planning [20]. The policy recommendations for introducing malaria vaccine RTS,S would be implemented in countries through the World Health Organization’s Immunization, Vaccines and Biologicals (IVB) (formerly the Expanded Programme on Immunization—EPI) [21]. Based on this, the case study was initiated with the following objective: to describe and analyse the Tanzanian population’s awareness and willingness to use malaria vaccines and to provide policymakers with evidence-based information on the best strategies to engage communities to manage the introduction of new vaccine and in this case malaria vaccine in Tanzania.

## Methods

### Study design and setting

The study was part of a large cross-sectional study covering all 23 regions of both Tanzania mainland and Zanzibar whose aim was to evaluate the success of Tanzanian Integrated Measles Campaign conducted during November 2011.

### Study sample size and sampling procedure

It was anticipated that the overall EPI coverage in the surveyed regions was estimated to be 85 % (the desired precision is ±5 % with 95 % confidence). Thirty clusters were sampled [22], and 9 women with children 0–11 months old per cluster were identified. A total of 6210 women with children 0–11 months old were recruited. For the purpose of this analysis, only 5502 women met the eligibility criteria and were included in the final analysis (n = 5502). The sampling procedure was based on 30-by-9 method and simple random sampling applied. The 30-by-9 method was a two-stage cluster sample. In the first stage, 30 clusters (corresponding to enumeration areas—EAs) were sampled by a probability proportion to size (PPS) strategy using the CSurvey software. In the second stage of sampling, nine eligible women with children 0–11 months old were selected within each EA.

Not all of the first nine households visited had an eligible child; therefore, more than nine households may have been visited. Similarly, fewer than nine households may have been selected if there was more than one eligible child per household. A sample of 30 enumeration clusters (villages) per region was surveyed; the minimum sample size was 270 mothers in each region. In each region, 30 clusters were visited and in each cluster, nine mothers with a child aged 0–11 months old were randomly selected and visited.

The following steps were followed:

- Within the regions, 30 EAs were selected using the PPS strategy.
- In each EA, nine eligible children were selected from households as follows:
- Go to the “centre” of the EA.
- Throw a pen to choose a random direction.
- Walk in that direction to identify the first household.
- Visit the first selected household and start to recruit the eligible children.
- After the first household visited, data collector moved to the “next household”, which was defined as the one whose front door was the closest to the just one visited.
- This process was continued until all nine eligible children were found/reached.

### Primary outcome and explanatory variables

The primary outcome variable was willingness to use a malaria vaccine; mothers were asked if they would like their children to receive malaria vaccine. The following explanatory variables of willingness to use a malaria vaccine were investigated: (1) awareness of the forthcoming malaria vaccine; mothers were asked if they ever heard about malaria vaccine. (2) Knowledge of the health benefits of vaccinating under-five children, mothers were assessed if they know malaria vaccine can prevent children from getting malaria, reduced disease infection and death or enhance good health. (3) Mothers to accept the mode of administering the malaria vaccine (require 2-3 jabs to receive full benefit). (4) Mothers to agree proposed schedule of given malaria vaccine at the same health facility and at the same time as other childhood vaccines. Other explanatory variables were ITNs ownership, EPI and measles vaccination.

### Data management and analysis

Data were double entered from data collection forms into a computer data file using Data Management System for Clinical Trials Software (DMSys) (Sigma soft International, Cincinnati, USA) [23]. Data were reviewed after the initial data entry to check for out-of-range responses, missing values, or inconsistent skip patterns; the original data collection sheets were reviewed to resolve any discrepancies or problems.

The data were analysed using STATA 11 standard edition software (StataCorp, TX, USA). The data were summarized using frequency tables and cross tabulation. Cross tabulation was done to assess the association between knowledge of the benefits of under-five vaccination and awareness of the forthcoming malaria vaccine; and between knowledge of the benefits of under-five vaccination and willingness to use a malaria vaccine. Categorical data was reported with numbers and percentage and their associated p values. Cross tabulation and Chi square was used to test association between variables in a two by two table. Fisher’s exact test was used to compare proportions in two by two tables where expected value in a cell was less than five. Univariate logistic regression was used to determine the magnitude of association for each exposure variable and outcome variable. Variables that showed association at a 0.25 significance level in univariate analysis were considered as candidates for the multivariate analysis. Multiple logistic regressions were used to determine the association between willingness and the primary exposure variable, while controlling for possible confounders. P values of less than or equal to 0.05 were considered significant.

### Ethical approval

The study was part of the Tanzanian Integrated Measles Campaign (IMC) survey in November 2011 and received ethical approval from the Institutional Review Boards of Ifakara Health Institute. Written informed consent was obtained from all participants prior to the start of the interviews.

## Results

Data was collected using structured questionnaires (Additional file 1) that assessed mothers of eligible children on awareness and willingness to use a malaria vaccine, health benefits of vaccinating under-five children, mode of administering malaria vaccine and its proposed schedule. The study involved 5502 mothers whom were asked about awareness of the forthcoming malaria vaccine, 11.8 % of participants in mainland Tanzania responded affirmatively, compared to 3.4 % in Zanzibar (Additional file 2); (p value <0.0001) (Table 1). 94.5 % of respondents were willing to take their children to get malaria vaccination, with a slight difference between mainland Tanzania (94.3 %) and Zanzibar (96.8 %) (Additional file 2); (p value = 0.0167) (Table 1).

**Table 1:** Comparison of perceived indicators of malaria vaccine between Zanzibar and Mainland, Tanzania

Perceived indicator	Zanzibar	Mainland	p value
Willingness	96.8 (511/528)	94.3 (4690/4974)	0.0167
Awareness	3.4 (18/528)	11.8 (589/4974)	<0.0001
Benefit	87.9 (464/528)	88.5 (4400/4974)	0.6917
Delivery mode	68.8 (363/528)	82.6 (4110/4974)	<0.0001
Proposed schedule	87.1 (460/528)	86.7 (4312/4974)	0.7816
ITN ownership	73.1 (386/528)	71.5 (3557/4974)	0.4380
Received EPI vaccines	90.8 (1316/1449)	83.8 (3353/4001)	<0.0001
Received measles vaccines	72.3 (1109/1534)	72.2 (5412/7498)	0.9378

Most (88.4 %) of the respondents reported knowing the benefits of vaccinating children under-five, with 88.5 %in mainland Tanzania and 87.9 % in Zanzibar (Additional file 3). The difference was not statistically significant (p value = 0.6917) (Table 1). The majority (81.3 %) of respondents reported accepting the mode of administering the malaria vaccine (2-3 jabs), with a high proportion (82.6 %) of acceptability among mainland Tanzanians than in Zanzibar (68.8 %) (Additional file 3); (p value <0.0001) (Table 1). Most (86.7 %) respondents would send their children for malaria vaccine according to the proposed schedule, with 86.7 % of respondents in mainland Tanzania and 87.1 % of respondents in Zanzibar (Additional file 3); the difference was not statistically significant (p value = 0.7816) (Table 1).

The proportion of respondents with knowledge of malaria prevention, mainly ITN ownership, was 71.7 % overall, and slightly higher in Zanzibar (73.1 %) as compared to mainland Tanzania (71.5 %); the difference was not significant (p value = 0.4380, Table 1). Respondents whose children received EPI vaccines were 84 % overall and significantly (90.8 %) in Zanzibar compared to mainland Tanzania (83.8 %). However, respondents whose children received EPI vaccines were statistically significant (p value <0.0001, Table 1). Overall, 72.2 % of respondents whose children received measles vaccines, respondents whose children received measles vaccines were similar between Zanzibar (72.3 %) and mainland Tanzania (72.2 %). The difference was not statistically significant (p value = 0.9378, Table 1).

## Discussion

Understanding community perceptions can help to identify issues to guide policy decisions for introducing the malaria vaccine. These findings are similar to studies documenting the need for early planning for new interventions, essential for policy decision making and relevant information can speed up the efforts to facilitate its implementation [20]. Understanding community perceptions of a malaria vaccine also helps to inform country programme managers responsible for National Malaria Control Programme (NMCP) and EPI/IVB priority setting and planning.

The low (11 %) level of awareness of the forthcoming malaria vaccine and high (94.5 %) willingness to use a malaria vaccine were similar to those found by Colon-Lopez and others for HPV vaccination in Puerto Rico [24], which also indicated low (28.3 %) level of awareness of and high (76.9 %) willingness to use HPV vaccines. Both findings come from settings where none of the study participants had been vaccinated. Despite low awareness about vaccine, mothers are still willing to vaccinate their children. This finding suggests that knowledge does not lead to achievement of vaccine coverage. This is quite consistent in many low-income countries. Thus, creating awareness of the malaria vaccine would be effective; although, currently understanding among respondents is low because the malaria vaccine is new and most people had not yet learned about it. Informing women about the malaria vaccine would likely increase women’s interest in their willingness to use a malaria vaccine. Creating awareness of malaria vaccine could reveal policy-related issues that, once addressed, could facilitate delivery of malaria interventions [9] and child vaccinations [25].

Willingness to use a malaria vaccine was compared to the knowledge of the benefits of vaccinating children under-five. This finding is consistent with others’ findings in Kenya and Ghana that showed wide spread knowledge of childhood vaccinations [12]; in Ghana, over 90 % of respondents understood that the malaria vaccine had benefits related to child vaccinations [12, 16, 25, 26]. Contrary to the study conducted when malaria vaccine efficacy results were not yet available, the level of willingness to use a malaria vaccine differed when respondents considered low efficacy results compared to other childhood vaccines [16, 27]. Knowledge of existing routine immunization schedules and benefits increased the level of willingness to use a malaria vaccine. The structure of the EPI programme in Tanzania is widely spread and accessible to the majority of Tanzanian women. As the malaria vaccine is expected to be delivered through the EPI programme, women would expect the vaccine’s benefits to be in line with those of other routine vaccinations. Therefore, informing women about the benefits of vaccinating children under-five is likely to increase women’s interest in the forthcoming malaria vaccine and their willingness to use it.

High acceptance of the mode of administering a malaria vaccine (2-3 jabs) according to the proposed schedule was similar to findings by Febir and others who showed that respondents were willing to receive vaccines in the form of injections, as most understood that “vaccines are injections given to children in their childhood to prevent occurrences of diseases” [16]. Contrary to Parvez and others, immunization injections were perceived to be painful procedures [28]. The injection method becomes a challenge when increasing numbers of injections as women become less willing to take their children for malaria vaccination. After the end of routine vaccination, parents might not take their children for additional vaccinations for a variety of reasons, including mothers’ competing priorities. Immunization clinics at health facilities and in informal areas (mobile clinics) can be good avenues for informing women about the malaria vaccine and for scheduling children for vaccination.

The strengths of the study include: larger sample size, representative sampling and combining data on awareness of the forthcoming malaria vaccine, willingness to use a malaria vaccine, knowledge of the benefits of vaccinating children under five, acceptability of the mode of administering the vaccine according to the proposed schedule, ITN ownership, and knowledge of EPI and measles vaccinations. The study had a number of limitations, including lack of demographic data and difficulty in determining acceptance of a malaria vaccine that is not yet available. It is likely that there are other reasons not covered by this study that account for some women’s lack of awareness of a forthcoming malaria vaccine and their unwillingness to use a malaria vaccine. For example, the vaccine may be accepted by the parents but still they do not take their children for vaccination due to distance, competing maternal priorities and lack of time.

## Conclusions and recommendations

Although mothers were highly unaware of a forthcoming malaria vaccine, they were very willing to use a malaria vaccine. Identifying regions with low awareness of malaria vaccine such as Iringa, Dar Es Salaam, Dodoma, Kagera, Kilimanjaro, Mbeya and Tanga would allow appropriate advocacy strategies to be planned and communication strategies to be developed before introducing the malaria vaccine in Tanzania. Malaria vaccine RTS,S will complement existing malaria interventions and be implemented through the IVB programme. The information generated by this study can aid policy makers as they plan and set priorities for introducing and implementing the malaria vaccine.

It is recommended that awareness of a potential malaria vaccine be created in the entire Tanzanian community, specifically among mothers who should be informed of both the benefits related to child vaccination and of the malaria vaccine. This could be accomplished by disseminating information to enhance maternal readiness for adopting malaria vaccination.

## Additional files

**Additional file 1.** Tool used to collect information on women’s behavioural aspects related to vaccine and malaria Vaccine. The data provided used for analysis of study on “Assessment of parental perception of malaria vaccine in Tanzania: A Case Study”.
**Additional file 2.** Percentage distribution of perceived awareness and willing to use malaria vaccine. The data provided represent the statistical analysis of awareness and willing to use malaria vaccine. Willingness to use malaria vaccine was higher in both Zanzibar and Tanzania mainland, however, awareness of malaria vaccine was low in the regions, with Zanzibar had the lowest understanding of awareness of malaria vaccine.
**Additional file 3.** Percentage distribution of perceived benefits, mode of administering malaria vaccine and acceptance of proposed schedule. The data provided represent the statistical analysis of benefits, mode of administering malaria vaccine and acceptance of proposed schedule. Majority of women in both Zanzibar and Tanzania mainland understand the benefits of vaccine and they are ready to send their children for vaccination on any proposed schedule. However, women from Tanzania mainland accept the mode of administration (2-3 jabs) more than women in Zanzibar.

## Abbreviations

EPI: Expanded Programme on Immunization
IMC: integrated measles campaign
IVB: immunization, vaccines and biologicals
IRS: indoor residual spraying
ITN: insecticide-treated nets
LLITNs: long-lasting insecticide-treated nets
TDHS: Tanzania Demographic and Health Survey
WHO: World Health Organization
