# Supplementary material for: Assessment of parental perception of malaria vaccine in Tanzania
Source: Malar J. 2015 Sep 17;14:355. doi: 10.1186/s12936-015-0889-7 (PMC4573291; doi:10.1186/s12936-015-0889-7)
Supplement: Supplementary file 1 — Additional file 1. Tool used to collect information on women’s behavioural aspects related to vaccine and malaria Vaccine. The data provided used for analysis of study on “Assessment of parental perception of malaria vaccine in Tanzania: A Case Study”. [file 12936_2015_889_MOESM1_ESM.docx]

**Additional file 1: Tool used to collect information on women’s behavioural aspects related to vaccine and malaria Vaccine**

A: General Information

| District | \|__\|__\| |
| --- | --- |
| Division | \|__\|__\|__\|__\|__\|__\|__\|__\|__\|__\|__\|__\|__\| |
| Ward | \|__\|__\|__\|__\|__\|__\|__\|__\|__\|__\|__\|__\|__\| |
| Village | \|__\|__\|__\|__\|__\|__\|__\|__\|__\|__\|__\|__\|__\| |
| Sub-village | \|__\|__\|__\|__\|__\|__\|__\|__\|__\|__\|__\|__\|__\| |
| Cluster number | \|__\|__\|__\|__\| |
| Household Number | \|__\|__\|__\| |
| Date of last born child (Date / Month / Year) | \|__\|__\|/\|__\|__\|/\|__\|__\|__\|__\| |
| Date of interview (Date / Month / Year) | \|__\|__\|/\|__\|__\|/\|__\|__\|__\|__\| |
| Time to start: Hours: Minutes | \|__\|__\|:\|__\|__\| |
| Time to finish: Hours*: Minutes* | \|__\|__\|:\|__\|__\| |
| Respondent Number \|__\|__\|__\|__\|__\|__\|__\|__\|__\|__\|__\|__\|__\|__\|__\|__\|__\|__\|__\| | |

**B: Status of the interview**

| 1 | What is the status of the interview? | (1) Complete |  |
| --- | --- | --- | --- |
|  |  | (2) Incomplete |  |
|  |  | (3) No interview |  |
| 2 | If incomplete or no interview, why? | (1) Verbal Consent not given |  |
|  |  | (2) Verbal Consent refused |  |
|  |  | (3) Other |  |
|  |  | (4) Specify_______________________ |  |

**C: GENERAL KNOWLEDGE**

| 1 | Apart from under-five child diseases with routine vaccination (TB, Polio, Measles and Hepatitis B, Diphtheria, Pertussis, Tetanus), which other health related problems do you consider to be serious in your community? (list up to five illnesses/diseases) | 1. ____________________________________ 2. ____________________________________ 3. ____________________________________ 4. ____________________________________ 5. ____________________________________ | |  |
| --- | --- | --- | --- | --- |
|  |  |  |  |  |
| 2 | Which illnesses/diseases you have mentioned above, do you propose them for vaccination? | 1. ____________________________________ 2. ____________________________________ 3. ____________________________________ 4. ____________________________________ | |  |
| 3 | Do you know of any benefits related to under-five child vaccination? | 1. Yes 2. No | |  |
| 4 | If yes, list them | 1. ___________________________________ 2. ____________________________________ 3. ____________________________________ 4. ____________________________________ | |  |
| 5 | What motivated you, for taking your child for vaccination? |  | |  |

**D: MALARIA**

| 1 | Do you know any malaria prevention strategies? | (1) Yes | | |  | |
| --- | --- | --- | --- | --- | --- | --- |
|  |  | (2) No | If **No** move to D3 | | | |
| 2 | If yes, list all methods of preventing malaria. *Please mention all that you can think of. (Check all that are mentioned). Which do you use at home?* | | | | | |
|  | **Methods of preventing malaria** | | | **Mentioned** | | **Home use** |
|  | ITN (bed-nets) use for children | | |  | |  |
|  | Residual spraying with insecticide | | |  | |  |
|  | Intermittent preventive treatment (IPTi) | | |  | |  |
|  | Drainage of mosquito breeding sites | | |  | |  |
|  | Block mosquito entry inside homes | | |  | |  |
|  | Plugging holes, closing windows/doors | | |  | |  |
|  | Burn things to create smoke inside house | | |  | |  |
|  | Cleaning environment around house | | |  | |  |
|  | Other____________________________________ | | |  | |  |
| 3 | Have you ever heard about malaria vaccine? | | | 1. Yes 2. No | | |
| 4 | If yes, where did you hear from? | | |  | | |
| 5 | Suppose a malaria vaccine could soon become available for under-five children in your community. I am now going to give you some information about the vaccine. After I read each statement, please tell me the extent to which you agree with the statements. | | | | | |
|  | A. Do you believe that malaria vaccination will bring any benefits related to under five child health? Yes/No/Not sure | | | | | |
|  | B. As Malaria vaccine will prevent cases of diseases; would you like your child to get the vaccine? Yes/No | | | | | |
|  | A. The vaccine can prevent many children from getting malaria.  5) Strongly agree 4) Agree 3) Not sure 2) Not agree 1) Strongly disagree | | | | | |
|  | B. The vaccine causes discomfort similar to other childhood vaccines  5) Strongly agree 4) Agree 3) Not sure 2) Not agree 1) Strongly disagree | | | | | |
|  | C. The vaccine will be given at the same health facility and at the same time as other childhood vaccines.  5) Strongly agree 4) Agree 3) Not sure 2) Not agree 1) Strongly disagree | | | | | |
|  | D. The malaria vaccine may require 2-3 jabs to receive full benefit.  5) Strongly agree 4) Agree 3) Not sure 2) Not agree 1) Strongly disagree | | | | | |
|  | E. Even though a child is vaccinated, s/he could still get malaria.  5) Strongly agree 4) Agree 3) Not sure 2) Not agree 1) Strongly disagree | | | | | |
|  | F. A vaccinated child who gets malaria will still need to receive treatment.  5) Strongly agree 4) Agree 3) Not sure 2) Not agree 1) Strongly disagree | | | | | |
|  | G. Even though a child is vaccinated, s/he still has to sleep under ITN.  5) Strongly agree 4) Agree 3) Not sure 2) Not agree 1) Strongly disagree | | | | | |
|  | H. The vaccine would prevent severe malaria in a vaccinated child.  5) Strongly agree 4) Agree 3) Not sure 2) Not agree 1) Strongly disagree | | | | | |

**E: PNEUMONIA AND DIARRHOEA**

| 1 | Do you consider pneumonia is serious disease among under-five children in your community? | (1) Yes | |  | |
| --- | --- | --- | --- | --- | --- |
|  |  | (2) No | |  | |
| 2 | Do you suggest pneumonia vaccine to be provided too? | (1) Yes |  | |  |
|  |  | (2) No | If **No**, then skip to E4 | |  |
| 3 | If yes, will you accept your child to be vaccinated with pneumococcal vaccine too? | (1) Yes  (2) No |  | |  |
| 4 | Do you consider diarrhoea is serious disease among under-five children in your community? | (1) Yes  (2) No |  | |  |
| 5 | Do you suggest diarrhoeal vaccine to be provided too? | (1) Yes  (2) No | If **No**, then Finish | |  |
| 6 | If yes, will you accept your child to be vaccinated with diarrheal vaccine too? | (1) Yes  (2) No |  | |  |
