# Supplementary material for: Assessment of parental perception of malaria vaccine in Tanzania
Source: Malar J. 2015 Sep 17;14:355. doi: 10.1186/s12936-015-0889-7 (PMC4573291; doi:10.1186/s12936-015-0889-7)
Supplement: Supplementary file 2 — Additional file 2. Percentage distribution of perceived awareness and willing to use malaria vaccine. The data provided represent the statistical analysis of awareness and willing to use malaria vaccine. Willingness to use malaria vaccine was higher in both Zanzibar and Tanzania mainland, however, awareness of malaria vaccine was low in the regions, with Zanzibar had the lowest understanding of awareness of malaria vaccine. [file 12936_2015_889_MOESM2_ESM.docx]

**Additional file 2: Percentage distribution of perceived awareness of and willing to use malaria vaccine**

| **Country / Region** | **Awareness** | **Willingness** |
| --- | --- | --- |
| **TANZANIA** | **11.0 (607/5502)** | **94.5 (5,201/5,502)** |
| ARUSHA | 12.5 (4/32) | 87.5 (28/32) |
| DAR ES SALAAM | 2.9 (7/239) | 97.1 (232/239) |
| DODOMA | 4.3 (13/306) | 89.5 (274/306) |
| IRINGA | 0.3 (1/291) | 89.0 (259/291 |
| KAGERA | 2.1 (5/243) | 94.2 (229/243) |
| KIGOMA | 5.9 (14/236) | 95.8 (226/236) |
| KILIMANJARO | 2.4 (6/253) | 95.3 (241/253) |
| LINDI | 6.1 (14/228) | 94.3 (215/228) |
| MANYARA | 68.4 (184/269) | 83.3 (224/269) |
| MARA | 33.1 (39/118) | 90.7 (107/118) |
| MBEYA | 3.7 (10/270) | 97.4 (263/270) |
| MOROGORO | 6.2 (15/242) | 97.9 (237/242) |
| MTWARA | 8.0 (19/237) | 97.5 (231/237) |
| MWANZA | 15.2 (43/283) | 98.9 (280/283) |
| PWANI | 6.8 (17/250) | 97.2 (243/250) |
| RUKWA | 16.5 (40/243) | 97.9 (238/243) |
| RUVUMA | 5.7 (15/262) | 90.1 (236/262) |
| SHINYANGA | 32.8 (82/250) | 95.2 (238/250) |
| SINGIDA | 11.1 (29/261) | 98.1 (256/261) |
| TABORA | 9.3 (26/280) | 97.1 (272/280) |
| TANGA | 3.3 (6/181) | 89.0 (161/181) |
| **MAINLAND** | **11.8 (589/4974)** | **94.3 (4,690/4,974)** |
| UNGUJA | 1.7 (5/298) | 97.7 (291/298) |
| PEMBA | 5.7 (13/230) | 95.7 (220/230) |
| **ZANZIBAR** | **3.4 (18/528)** | **96.8 (511/528)** |
