# Supplementary material for: Assessment of parental perception of malaria vaccine in Tanzania
Source: Malar J. 2015 Sep 17;14:355. doi: 10.1186/s12936-015-0889-7 (PMC4573291; doi:10.1186/s12936-015-0889-7)
Supplement: Supplementary file 3 — Additional file 3. Percentage distribution of perceived benefits, mode of administering malaria vaccine and acceptance of proposed schedule. The data provided represent the statistical analysis of benefits, mode of administering malaria vaccine and acceptance of proposed schedule. Majority of women in both Zanzibar and Tanzania mainland understand the benefits of vaccine and they are ready to send their children for vaccination on any proposed schedule. However, women from Tanzania mainland accept the mode of administration (2-3 jabs) more than women in Zanzibar. [file 12936_2015_889_MOESM3_ESM.docx]

**Additional file 3: Percentage distribution of perceived benefits, mode of administering malaria vaccine and acceptance of proposed schedule**

| **Country / Region** | **Benefits** | **Mode of administering** | **Proposed schedule** |
| --- | --- | --- | --- |
| **TANZANIA** | **88.4 (4,864/5502)** | **81.3 (4,473/5,502)** | **86.7 (4,772/5502)** |
| ARUSHA | 75.0 (24/32) | 62.5 (20/32) | 87.5 (28/32) |
| DAR ES SALAAM | 94.1 (225/239) | 83.3 (199/239) | 90.8 (217/239) |
| DODOMA | 86.9 (266/306) | 85.0 (260/306) | 90.2 (276/306) |
| IRINGA | 58.1 (169/291) | 83.2 (242/291) | 78.0 (227/291) |
| KAGERA | 94.7 (230/243) | 87.2 (212/243) | 92.6 (225/243) |
| KIGOMA | 86.4 (204/236) | 88.1 (208/236) | 96.2 (227/236) |
| KILIMANJARO | 92.5 (234/253) | 90.5 (229/253) | 91.7 (232/253) |
| LINDI | 95.2 (217/228) | 90.4 (206/228) | 93.4 (213/228) |
| MANYARA | 72.9 (196/269) | 75.8 (204/269) | 77.0 (207/269) |
| MARA | 89.0 (105/118) | 85.6 (101/118) | 86.4 (102/118) |
| MBEYA | 97.0 (262/270) | 90.0 (243/270) | 93.0 (251/270) |
| MOROGORO | 92.6 (224/242) | 73.1 (117/242) | 93.4 (226/242) |
| MTWARA | 94.1 (223/242) | 78.9 (187/237) | 94.9 (225/237) |
| MWANZA | 98.6 (279/283) | 93.3 (264/283) | 71.0 (201/283) |
| PWANI | 92.0 (230/250) | 88.0 (220/250) | 82.0 (205/250) |
| RUKWA | 97.5 (237/243) | 97.1 (236/243) | 95.9 (233/243) |
| RUVUMA | 86.6 (227/262) | 38.9 (102/262) | 84.0 (220/262) |
| SHINYANGA | 91.2 (228/250) | 66.8 (167/250) | 60.8 (152/250) |
| SINGIDA | 90.4 (236/261) | 83.9 (219/261) | 92.7 (242/261) |
| TABORA | 96.1 (269/280) | 95.4 (267/280) | 94.3 (264/280) |
| TANGA | 63.5 (115/181) | 81.2 (147/181) | 76.8 (139/181) |
| **MAINLAND** | **88.5 (4400/4974)** | **82.6 (4,110/4,974)** | **86.7 (4,312/4,974)** |
| UNGUJA | 88.6 (264/298) | 75.2 (224/298) | 86.6 (258/298) |
| PEMBA | 87.0 (200/230) | 60.4 (139/230) | 87.8 (202/230) |
| **ZANZIBAR** | **87.9 (464/528)** | **68.8 (363/528)** | **87.1 (460/528)** |
